# Compromised global embryonic transcriptome associated with advanced maternal age

**DOI:** 10.1007/s10815-019-01438-5

**Published:** 2019-04-25

**Authors:** Blair R. McCallie, Jason C. Parks, G. Devon Trahan, Kenneth L. Jones, Breanne D. Coate, Darren K. Griffin, William B. Schoolcraft, Mandy G. Katz-Jaffe

**Affiliations:** 1 Colorado Center for Reproductive Medicine, Lone Tree, CO USA; 2 University of Kent, School of Biosciences, Canterbury, CT2 7NZ UK; 3 University of Colorado, Anschutz Medical Campus, Aurora, CO USA; 4 Oregon Reproductive Medicine, Portland, OR USA

**Keywords:** Advanced maternal age, Human blastocyst, Transcriptome, Gene expression

## Abstract

**Purpose:**

To investigate the global transcriptome and associated embryonic molecular networks impacted with advanced maternal age (AMA).

**Methods:**

Blastocysts derived from donor oocyte IVF cycles with no male factor infertility (< 30 years of age) and AMA blastocysts (≥ 42 years) with no other significant female factor infertility or male factor infertility were collected with informed patient consent. RNA sequencing libraries were prepared using the SMARTer® Ultra® Low Kit (Clontech Laboratories) and sequenced on the Illumina HiSEQ 4000. Bioinformatics included Ingenuity® Pathway Analysis (Qiagen) with ViiA™ 7 qPCR utilized for gene expression validation (Applied Biosystems).

**Results:**

A total of 2688 significant differentially expressed transcripts were identified to distinguish the AMA blastocysts from young, donor controls. 2551 (95%) of these displayed decreased transcription in the blastocysts from older women. Pathway analysis revealed three altered molecular signaling networks known to be critical for embryo and fetal development: CREBBP, ESR1, and SP1. Validation of genes within these networks confirmed the global decreased transcription observed in AMA blastocysts (*P* < 0.05).

**Conclusions:**

A significant, overall decreased global transcriptome was observed in blastocysts from AMA women. The ESR1/SP1/CREBBP pathway, in particular, was found to be a highly significant upstream regulator impacting biological processes that are vital during embryonic patterning and pre-implantation development. These results provide evidence that AMA embryos are compromised on a cell signaling level which can repress the embryo’s ability to proliferate and implant, contributing to a deterioration of reproductive outcomes.

**Electronic supplementary material:**

The online version of this article (10.1007/s10815-019-01438-5) contains supplementary material, which is available to authorized users.

## Introduction

The effects of female aging on fertility are well described and impact, among others, ovarian reserve, oocyte quality, and pregnancy complications [1]. Chromosome aneuploidy is a significant contributor to infertility, with maternal age being the greatest risk factor [2]. Meiotic events occurring during oogenesis, particularly the prolonged arrest in dictyate, increase the susceptibility of chromosome segregation errors and this is observed in the oocytes from older women [3]. The association between increasing maternal age and the frequency of chromosome aneuploidy in human conception, including Down’s syndrome, has been extensively documented [4]. This leads to an increased risk of spontaneous abortion as women age, with more than half of all pregnancies resulting in a fetal loss by the time a woman reaches 42 years of age [5]. In fact, 75% of spontaneous miscarriages in women 35 years and older are the direct result of chromosome anomalies, compared to 50% in mothers younger than 35 years of age [6]. Infertile women of advanced maternal age (AMA) can utilize assisted reproductive technologies (ART) and pre-implantation genetic testing for aneuploidy (PGT-A) to selectively transfer euploid embryos with successful clinical outcomes. Nevertheless, these women do see a decrease in live birth rates compared to their younger counterparts, indicating a reduced implantation potential independent of chromosome constitution.

Oogenesis is a process that involves a complex series of nuclear and cytoplasmic events that prepare the oocyte for fertilization and initiate pre-implantation embryo development until the activation of the embryonic genome [7, 8]. The quality of the oocyte will determine an embryo’s developmental potential and an aged oocyte can have dysfunctions in the cellular organelles including, among others, the endoplasmic reticulum and mitochondria which play a role in Ca^2+^ storage and absorption, culminating in apoptosis [9–11]. This storage and redistribution of calcium by the endoplasmic reticulum is responsible for cell activation during fertilization and can affect embryo development and implantation [12]. Mitochondria are essential for oocyte maturation, fertilization, and development since they act as the major source of ATP during pre-implantation embryonic development [13]. Damage to mitochondria can also cause increased production of reactive oxygen species (ROS), via oxidative phosphorylation during ATP production, and accumulate over time to expose the aged oocyte to oxidative stress [14]. Taken together, this can promote the aging process by negatively influencing cell signaling pathways that are involved in proliferation, differentiation, and apoptosis which then result in DNA damage or developmental arrest [15, 16].

Aging can also impact epigenetic factors, specifically histone modifications, which are essential for oocyte development [17]. There are several forms of modifications at the histone amino termini including methylation, acetylation, phosphorylation, and ubiquitination, all of which play important roles in cell cycle progression, DNA replication and repair, and transcriptional activity [18–20]. Histone acetylation in particular is critical for these cellular functions, as well as regulating chromosome segregation and various chromatin-based processes [21]. In mammalian oocytes, histones are deacetylated by histone deacetylase (HDAC) genes during meiosis and inhibitions to HDAC activity have been reported to induce aneuploidy and early embryonic death in mice [21, 22].

There is currently limited knowledge of how advanced maternal aging impacts the developmental competence of an embryo on a molecular level. More recently, a study looked at the effects of parental age on downstream gene expression in human blastocysts and found that maternal age had a significant impact on changes in the blastocyst transcriptome with more than 800 genes having reduced expression as maternal age increased (ranging from 31 to 41 years of age) [23]. Among these downregulated genes, several were considered to be important for meiotic chromosomal segregation, cell cycle control, and embryo growth and implantation.

The aim of our study was to elucidate the cellular transcriptome of human blastocysts from women of considerable advanced maternal age (≥ 42 years), to further our understanding of oocyte aging and its impact on embryonic competence and reproductive success. This knowledge will provide a valuable molecular explanation, independent of chromosome constitution, for the lower success rates observed in this patient population.

## Materials and methods

### Human blastocysts

Surplus, cryopreserved, transferrable quality (grade ≥ 3BB) human blastocysts were fertilized using intracytoplasmic sperm injection and sequentially cultured under low oxygen conditions prior to being donated to research with Institutional Review Board approval and patient consent: young, oocyte donor control with no male factor infertility (DC < 30 years; *n* = 12 from 6 different patients), and advanced maternal age with no male factor or other significant female factor infertility (AMA ≥ 42 years old; *n* = 12 from 12 different patients). Blastocysts were identified by PGT-A to be void of autosomal chromosomal aneuploidies and were warmed following previously published vitrification protocols [24]. All fathers were ≤ 48 years of age and therefore not considered to be advanced paternal age.

### RNA isolation

RNA was isolated from individual blastocysts using the PicoPure™ RNA Isolation Kit (Applied Biosystems, Foster City, CA) with minor modifications to the manufacturer’s protocol. Briefly, blastocysts were lysed at 42 °C for 30 min in 10 μl of extraction buffer. One volume of 70% EtOH was mixed with each sample prior to loading onto a pre-conditioned purification column. Each sample was on-column deoxyribonuclease treated at room temperature for 15 min (Qiagen, Germantown, MD). After several washes, RNA was eluted in 20 μl of elution buffer.

### RNA sequencing and bioinformatic analysis

The entirety of the purified RNA from each blastocyst (*n* = 12, 6 from each group) was utilized to prepare sequencing libraries using the SMARTer® Ultra® Low Kit (Clontech Laboratories, Fremont, CA) following manufacturer’s instructions and then sequenced on the HiSEQ 4000 (Illumina, San Diego, CA) as single pass 50 bp reads. Derived sequences were processed by performing quality checks and normalization on each read and then mapping them to the human genome (build GRCh38) using Trimmomatic v0.36 [25], GMAP-GSNAP v2014-12-17 [26], SAMtools v1.5 [27], and Cufflinks v2.2.1 [28]. Transcripts with no reads across all samples, as well as transcripts in the bottom quintile based on mean expression across all samples, were excluded from analysis. A log transformation was performed prior to statistical analysis which included a 2-sample, 2-sided, independent Student’s *t* test (significance at Q < 0.05). The false discovery rate was then adjusted using the Benjamini-Hochberg procedure and the expression log ratio was used for each differentially expressed transcript. Pathway analysis was performed on differentially expressed transcripts using Ingenuity Pathway Analysis (Qiagen). The density of gene start site positions was quantified by performing a kernel density estimation (KDE) for each chromosome using the distplot function from the Seaborn v0.8.1 package for Python (www.python.org). Bandwidth for the KDE was set manually for chromosome 20 and scaled linearly for all other chromosomes based on chromosome length. To determine whether the KDE for the differentially expressed transcripts was a common occurrence, a 95% percentile interval was constructed. A total of 2699 gene start sites were randomly selected (11 of which were start sites for identical genes) and a KDE was performed. This process was repeated 1000 times to obtain 1000 distributions. For each point where the KDE was calculated, values were obtained for the 97.5th percentile and the 2.5th percentile. These values were then used to construct an interval that would contain 95% of the randomly generated distributions.

### Sequencing validation and analysis

Sequencing validation on genes of interest was completed with isolated RNA from additional blastocysts (*n* = 12, 6 from each group). Reverse transcription was performed using the High-Capacity cDNA Reverse Transcription Kit (Applied Biosystems) and cDNA was diluted (1:4) in 1X Tris-EDTA buffer prior to performing quantitative reverse transcription PCR (RT-qPCR) on the QuantStudio 5 Real-Time PCR System (Applied Biosystems). Three microliters of diluted cDNA was combined with 5 μM primer mix and *Power* SYBR™ Green PCR Master Mix (Applied Biosystems) in a 15 μl final volume and amplified under the following thermal cycling conditions: 95 °C for 10 min followed by 40 cycles at 95 °C for 15 s and 60 °C for 1 min, and a melt curve stage at 95 °C for 15 s, 60 °C for 1 min, and 95 °C for 15 s. Each sample was run in duplicate for 11 genes of interest (ALK, TNFRSF10A, TSPAN9, CCND3, GNAS, LTBP3, MAPK8IP1, NDRG1, SREBF1, EPSTI1, TLE2) and analyzed compared to the expression of three, stable, housekeeping genes (GAPDH, PPIA, RPL19). Statistical analysis was performed using REST 2009 software (Qiagen) which uses PCR efficiencies and mean crossing point deviation between the sample and control groups to test for significance by a Pair Wise Fixed Reallocation Randomisation Test© (significance at *P* < 0.05) [29]. The most consistent housekeeping gene was selected for normalization (PPIA).

### Ethical approval

All participants provided written informed consent and this study was approved by the Western Institutional Review Board (protocol no. 20140458).

## Results

### RNA sequencing

**Fig. 1:**
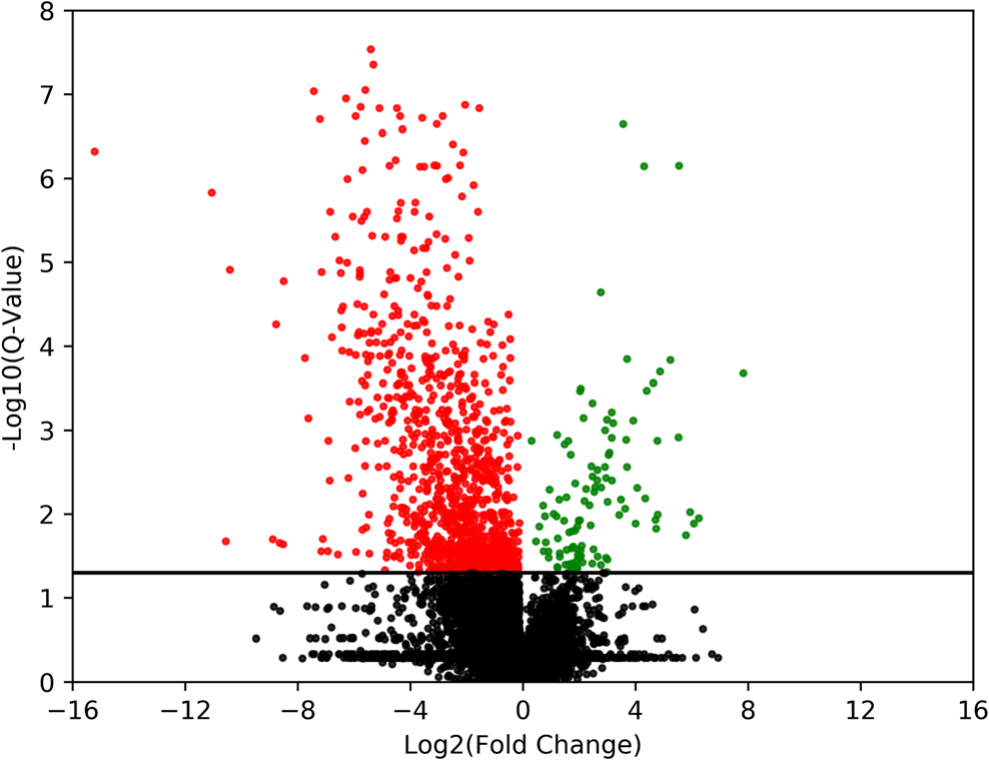
Differentially expressed transcripts in AMA blastocysts compared to donor control (DC). The y-axis corresponds to the mean expression value of Log_10_ (Q-value) and the x-axis displays the Log2 (fold change) value. Red dots represent transcripts with statistically significant negative fold changes in AMA blastocysts (Q < 0.05), whereas the green dots represent transcripts with statistically significant positive fold changes in AMA blastocysts (Q < 0.05). Black dots denote genes that were not significantly altered

RNA sequencing data was collected and an average of 46.9 million reads were acquired for each blastocyst. Quality filters were applied to all samples to remove reads with poor sequencing quality resulting in 91% of the reads passing these filters and proceeding for alignment to the human reference genome (Supplementary Table 1). One sample from the DC group was eliminated from further analysis due to poor quality. Expression intensity was calculated using RPKM method and only transcripts with a mean expression of 0.05 or greater were considered for analysis, resulting in 26,489 Ensembl IDs expressed between the two sample sets. A FDR adjusted *p* value was used to determine significance and reduce false positives. Additional quality control was performed to identify and remove any outliers and overly abundant transcripts to reduce normalization artifact. In total, 2688 (10%) of the expressed transcripts were considered significantly differentially transcribed in AMA blastocysts compared to DC with 2551 of these having decreased expression (95%) and 137 displaying increased transcription (5%) (Supplementary Table 2; Q < 0.05). When considering all transcripts analyzed (including those that were non-significant), 70% displayed decreased expression in the AMA sample set, revealing an overall global decrease in transcription in the blastocysts from this group. Volcano plot analysis depicts this trend with the vast majority of significantly differentially expressed transcripts having a negative fold change in AMA blastocysts (Fig. 1).

**Fig. 2:**
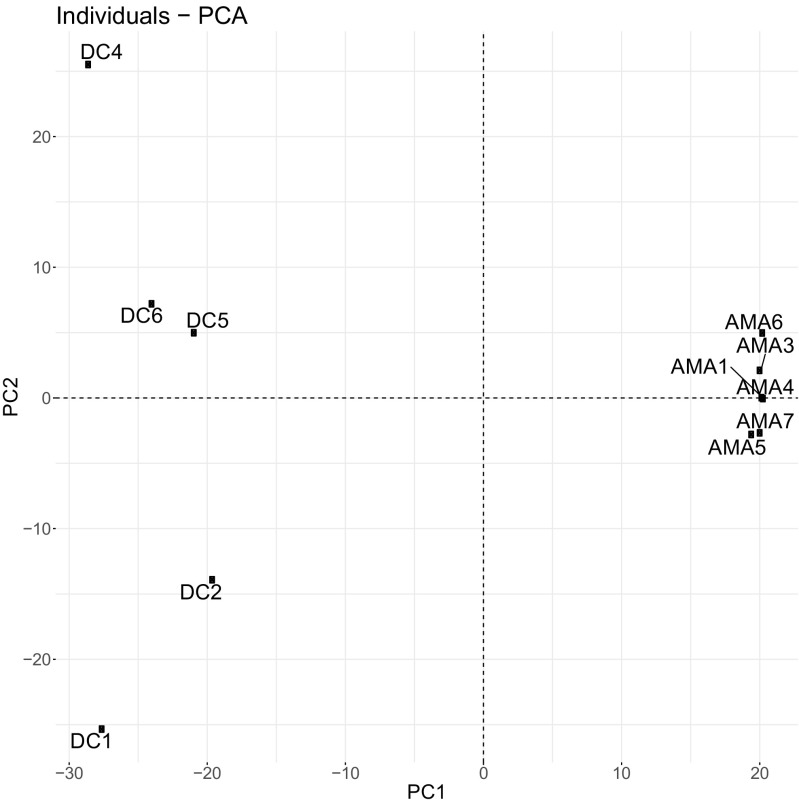
Principle component analysis (PCA) depicting a clear separation of AMA blastocysts from donor controls (DC)

**Fig. 3:**
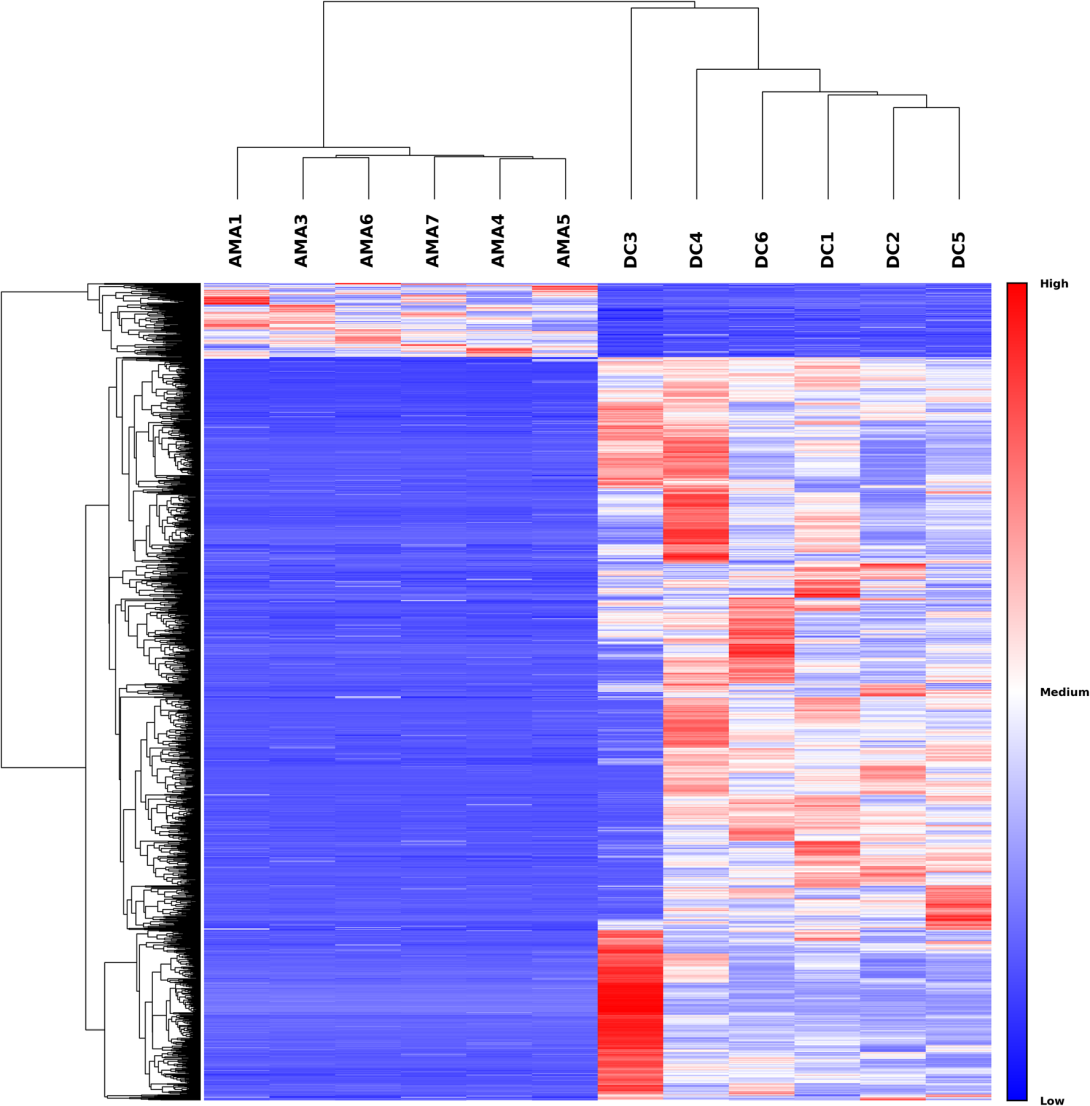
Unsupervised hierarchical clustering of differentially expressed transcripts in AMA blastocysts vs. donor control (DC) showing a distinct separation between the two groups. Red denotes upregulation while purple (change from purple to blue if new attachement is used) blue represents downregulation (Q < 0.05)

Principle component analysis (PCA) was performed to reveal global differences between the sample groups. Two distinct sets were identified using PCA, with the AMA blastocysts grouping together but separately from the grouped DC blastocysts, indicating uniformity and low biological variability among samples within each group (Fig. 2). Unsupervised hierarchical clustering analysis also distinguished the two groups, with each having uniquely different transcription patterns and branching separately (Fig. 3). The uniformity observed in the AMA blastocyst transcriptome reflects a strong phenotype for this sample group. Examination of the gene density on individual chromosomes revealed a significantly higher number (> 30%; Q < 0.05) of differentially expressed transcripts localized at the telomeric regions for 9 chromosomes (4, 9, 11, 16, 17, 19, 20, 21, 22) (Supplementary Figure 1). Over a quarter (28%) of the differentially expressed transcripts in this study were found within 10 Mb of the telomeric regions at both chromosome ends.

**Fig. 4:**
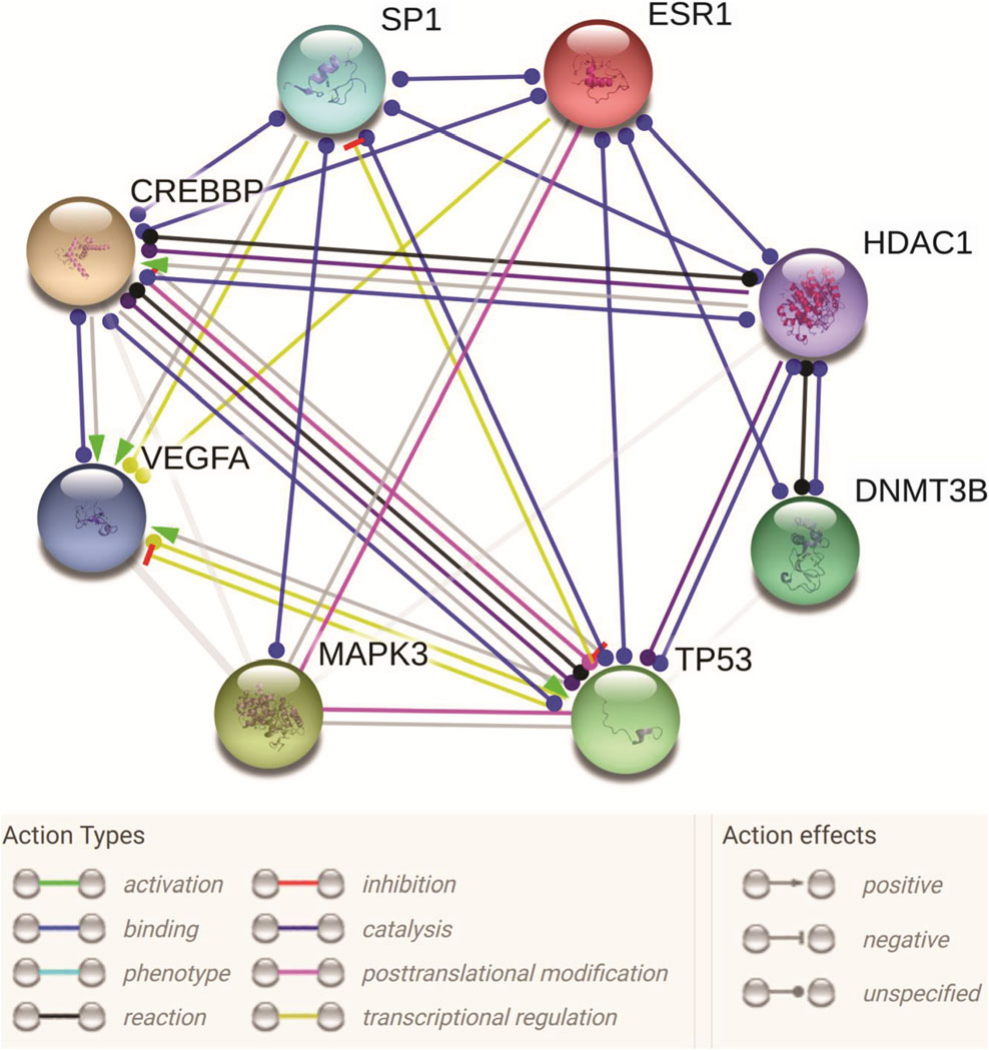
Molecular signaling network predicted to be associated with the global decrease in transcription observed in AMA blastocysts compared to donor control (DC) blastocysts

Canonical pathway analysis (IPA®) of the 2688 significantly altered transcripts revealed hits for G protein–coupled receptor signaling, calcium signaling, AMPK signaling, and gap junction signaling, among others (Table 1). Expanded pathway analysis of these statistically significant transcripts revealed a set of interacting molecular signaling networks and upstream regulators important for embryo development which included SP1, ESR1, HDAC1, DMNT3B, TP53, MAPK3, VEGFA, and CREBBP (Fig. 4). A Regulator Effects analytic (IPA®) was performed to identify the top 5 upstream regulators driving the observed global decreased transcription in AMA blastocysts. Altered pathways that are predicted to work with other molecules and lead to changes in downstream RNA expression included pathways involved in invasion, transmission and activation of cells, cell movement, and organismal death in blastocysts from women of advanced maternal age (Supplementary Figure 2).

**Table 1:** Key canonical pathways that are predicted to be altered in AMA blastocysts vs. donor control (DC) blastocysts (*P* < 0.05)

Ingenuity canonical pathways	*p* value	Importance	Molecules
G protein–coupled receptor (GPCR) signaling	7.41E-08	Required for normal embryonic development	PDE6A,HTR1B,MAP2K2,ADCY5,FGFR4,HTR1E,HTR7,GRM6,IRS2, ADRA1B,PIK3C2B,PDE2A,APLNR,OPRM1,GR8,SSTR3,RAPGEF3,RAP1A,GRM7,RAP1GAP,PDE1B,GRM2,CAMK4,HTR4,PTGER3,NFKBIE,TBXA2R, CHRM4,PIK3R5,PDE4A,RAPGEF4,RGS12,HRH3,PRKAG1,OPRL1,EP300, ADRB1,PDE3B,PRKCE,PIK3R2,DRD3,CAMK2B,SRC,HTR6,ADCY2,GNAS, NPY1R,ADCY6,GNAQ,OPRD1,GPER1,GLP1R,OPRK1,PRKCB
Gαi signaling	1.05E-06	Inhibits the production of cAMP from ATP	GRM2,PTGER3,TBXA2R,CHRM4,RGS12,HRH3,PRKAG1,GNG7,OPRL1, HTR1B, ADCY5,HTR1E,GRM6,DRD3,RALGDS,SRC,ADCY2,APLNR,GNAS, OPRM1,GRM8,NPY1R,SSTR3,ADCY6,RAP1A,GRM7,OPRD1,RAP1GAP, OPRK1
cAMP-mediated signaling	2.34E-06	GPCR-triggered signaling cascade used in cell communication	GRM2,CAMK4,HTR4,PTGER3,CHRM4,TBXA2R,PDE4A,RAPGEF4,RGS12, HRH3, OPRL1,EP300,PDE6A,HTR1B,ADRB1,MAP2K2,PDE3B,ADCY5, HTR1E,HTR7, GRM6,DRD3,CAMK2B,HTR6,SRC,PDE2A,ADCY2,GNAS, APLNR,OPRM1,NPY1R, GRM8,SSTR3,ADCY6,RAPGEF3,RAP1A,GRM7, OPRD1,GPER1,RAP1GAP,GLP1R,PDE1B,OPRK1
PKCθ signaling in T lymphocytes	3.39E-04	Immune response, promotes activation-induced T cell death	CD247,CACNA1S,MAP3K11,HLA-A,NFKBIE,PIK3R5, HLA-DQB1,NFATC1,LCK,CACNA1E,FGFR4,IRS2, PIK3R2,CACNA2D3,MAP3K2,CAMK2B,PIK3C2B,CACNB1,MAP3K6, CHP1,PLCG1,CACNA1C,NFATC4, RAP1A,CACNA1A,VAV3,ZAP70, NFATC2,HLA-DOB
Calcium signaling	3.80E-04	Increase in cytosolic Ca2+ culminates in the regulation of transcription factors including NFAT, CREB, and HDACs. Ca2+ signaling is associated with events during embryogenesis	CHRNA1,CACNA1S,CAMK4,TNNI2,MYL2,GRIN2D,GRIA1,TNNT2, PRKAG1,NFATC1, EP300,HDAC6,CACNA1E,RYR1,CACNA2D3,CAMK2B, CACNB1,CHRNA4,CHP1, CACNA1C,TNNI3,CHRNA10,NFATC4,CHRND, RAP1A,PNCK,CACNA1A,GRIN3A, MICU1,HDAC3,ATP2B3,CAMKK1, NFATC2,CAMKK2
AMPK signaling	4.47E-04	Inhibits key enzymes of ATP consuming pathways and induces pathways that generate ATP, stimulates fatty acid oxidation	CHRNA1,RAB9B,CHRM4,PIK3R5,LIPE,CFTR,PFKL,MAPK13,CCND1, MAPK11, PRKAG1,EP300,ADRB1,CRTC2,TBC1D1,FGFR4,TSC2,PPM1L, IRS2,PPP2R2C, CPT1C,PIK3R2,ADRA1B,SRC,PIK3C2B,RAB27A,CHRNA4, ACACB,CPT1A,GNAS, AK3,CHRNA10,CHRND,FOXO6,CAMKK2
Gap junction signaling	5.01E-04	Critically important in regulating embryonic development	GRIA1,PIK3R5,NOTUM,EGF,PRKAG1,PLCE1,ADRB1,MAP2K2,ADCY5, FGFR4,TUBA3C/TUBA3D,PRKCE,IRS2,PIK3R2,TUBA3E,ACTG2,GJB2, MAP3K2,SRC,PIK3C2B,GJA1,ADCY2,GNAS,GUCY1A1,TJP1,GNAQ, ADCY6,PLCG1,PLCL2,RAP1A,GJC1,GUCY1A2,PRKCB
Phospholipase C signaling	5.75E-04	Plays a role in embryonic development including cell migration, proliferation, and differentiation	CD247,PLD2,CAMK4,MYL2,ARHGEF1,NFATC1,GNG7,EP300,TGM2, HDAC6,LCK,IGHG3,PLCE1,MAP2K2,RHOD,ADCY5,PRKCE,RALGDS, SRC,ADCY2,GNAS,CHP1,ADCY6,GNAQ,PLCG1,RAPGEF3,NFATC4, RAP1A, PLD1,PLA2G6,PLA2G2E,HDAC3,SYK,PLA2G4B,ZAP70,NFATC2, ARHGEF10,PRKCB
GNRH signaling	6.31E-04	Plays a critical role in blastocyst formation	CACNA1S,MAP3K11,CAMK4,MAPK13,MAPK11,PRKAG1,GNG7,EP300, CACNA1E,MAP2K2,ADCY5,PRKCE,CACNA2D3,CAMK2B,MAP3K2,SRC, CACNB1,ADCY2,GNAS,PAK6,MAP3K6,GNAQ,ADCY6,CACNA1C,RAP1A, CACNA1A, MAPK10,PRKCB,GNRHR
Th1 pathway	9.12E-04	Th1:Th2 balance plays an important role in successful pregnancy maintenance and is associated with a decline in responsiveness to Th1 activation during aging	CD247,PIK3C2B,DLL1,CCR5,HLA-A,KLRD1,TYK2,PIK3R5,NFATC4, HLA-DQB1, CD8A,NFATC1, ITGB2,FGFR4,APH1B,NFATC2, HLA-DOB,IRS2,IL27RA, PIK3R2,JAK3, HLA-DPA1,IFNA1/IFNA13,ICOSLG/LOC102723996

### Sequencing validation

**Fig. 5:**
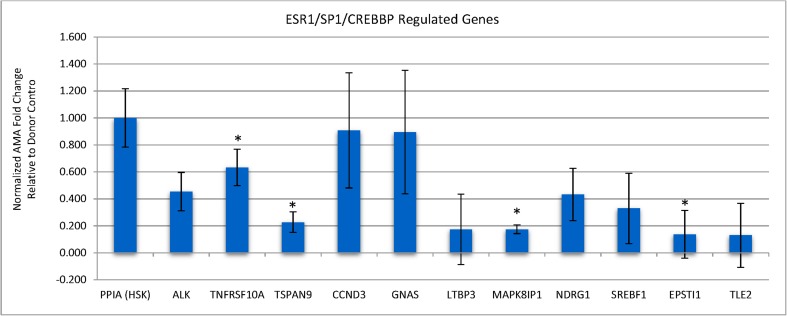
RT-qPCR validation of genes within the ESR1/SP1/CREBBP molecular signaling network that displayed reduced expression in AMA blastocysts vs. donor control (DC) blastocysts (*P < 0.05)

Genes chosen for validation utilizing RT-qPCR focused on the transcription ligand-dependent activation of ESR1/SP/CREBBP pathway as it was observed to be a highly significant upstream regulator important for a variety of biological processes including cell cycle regulation, proliferation, and apoptosis. In total, 62 genes regulated by the ESR1 network were found to be significantly decreased in AMA blastocysts, as well as 56 genes regulated by SP1, and 41 genes regulated by CREBBP (*P* < 0.05). ESR1-regulated genes chosen for validation included ALK, TNFRSF10A, and TSPAN9. SP1-regulated genes examined for validation were CCND3, GNAS, LTBP3, MAPK8IP1, NDRG1, and SREBF1. Finally, CREBBP-regulated genes tested included EPSTI1 and TLE2. All genes displayed a trend towards reduced expression in additional AMA blastocysts tested by RT-qPCR, compared to DC, confirming our observations from the RNA sequencing analysis (Fig. 5). TNFRSF10A and TSPAN9, which are regulated by ESR1, were significantly reduced in the blastocysts from older women (fold change = 0.63 and 0.23 respectively; *P* < 0.05; Fig. 5). MAPK8IP1 which is regulated by SP1 was also significantly reduced in AMA blastocysts (fold change = 0.17; *P* < 0.05; Fig. 5). EPSTI1, regulated by CREBBP, was the final gene that displayed a significant reduction in gene expression among the AMA group (fold change = 0.14; *P* < 0.05; Fig. 5).

## Discussion

Transcriptome analysis of blastocysts from women of advanced maternal age revealed a significant, global downregulation of gene expression. This altered transcriptome could compromise future embryonic developmental competence, explaining the decreased implantation and live birth rates observed in this patient population, following a euploid embryo transfer.

The ESR1/SP1/CREBBP pathway was predicted as a highly significant upstream regulator driving the observed widespread downregulation in embryonic gene transcription associated with AMA. ESR1 is a ligand-activated transcription factor that can stimulate transcription directly or by activation of other transcription factors in a ligand-dependent manner [30]. For ESR1 to function, it requires the recruitment of coactivators such as SP1 which, in turn, recruit secondary coactivators including CREBBP that promote chromatin remodeling and facilitate the activation of transcription [31, 32]. Cancer studies have shown that when ESR1 is under-expressed, it fails to activate SP1, resulting in decreased expression of downstream genes that regulate a variety of processes including cell cycle regulation, proliferation, and apoptosis [31]. Furthermore, CREBBP has been previously shown to be essential for mouse embryogenesis [33].

The ESR1-regulated gene TNFRSF10A that displayed reduced expression with advanced maternal age has been implicated in the process of cellular apoptosis. Programmed cell death plays an important role in gamete maturation and embryo development. Both the inner cell mass and the trophectoderm have active apoptotic pathways that are required for ongoing, successful development. Disruptions to the apoptotic pathway can compromise future growth and result in reproductive failure [34]. TSPAN9, another decreased ESR1-regulated gene in AMA blastocysts, plays a role in the regulation of cell development, activation, growth, and motility. Earlier studies have shown that TSPAN9 is localized to the plasma membrane and cytoplasm, and could be required for maintaining the early endosome membrane [35]. The endosome functions as a signaling center for various ligand-receptor systems that have critical roles in embryonic patterning, particularly in the peri-gastrulation stage of embryo development [36]. Since gastrulation occurs shortly after implantation, any signaling errors in the embryo during this critical time would likely negatively impact reproductive success. A previous study utilizing *C. elegans* found that a number of tetraspanins, including TSP-21 (orthologous to human TSPAN9), promote BMP signaling in postembryonic development [37]. BMP has been shown to be important for pre-implantation development and gastrulation in mouse embryos [38]. Both BMP1 and BMP7 were observed in this study to be significantly reduced in expression with advanced maternal age. BMP1 has also been shown to promote oocyte maturation and early embryo development in porcine embryos that were fertilized in vitro [39]. Several studies have displayed the importance of BMP7 for not only the transformation of the endometrium into a receptive state, but also for decidualization, and placental/embryonic development [40–42].

The SP1-regulated gene, MAPK8IP1, was also significantly reduced in blastocysts derived from AMA women. This protein prevents MAPK8-mediated activation of transcription factors and plays a key role in cell signaling. It is also involved in the JNK signaling pathway and alterations can induce changes to cellular physiology, including cell death [43]. The JNK pathway has been previously reported to be required for the 8–16 cell stage of embryonic development to the blastocyst stage [44]. Additionally, Thompson et al. found that targeted disruption of Mapk8ip1 in the mouse embryo resulted in embryonic death prior to blastocyst implantation [45].

The CREBBP-regulated gene, EPSTI1, is known to promote tumor invasion and metastasis. While widely known for its role in invasive breast carcinomas, this gene has also been found to be crucial in endometrial remodeling prior to embryo attachment [46]. The trophoblast cells of an early embryo rapidly proliferate and attempt to invade the endometrial decidua, later proliferating and migrating into the uterine wall to anchor the placenta [47]. These actions are extraordinarily similar to cancer cells with their capacity for proliferation and migration [47]. The reduced expression observed in EPSTI1 with advanced maternal age is therefore likely to not only reduce the ability of the pre-implantation embryo to proliferate and invade the endometrium, but also hinder the molecular cross-talk at the fetal-maternal interface, thereby preventing implantation.

The overall global decrease in transcription observed in AMA blastocysts also impacted chromatin structure with two histone deacetylase genes shown to have reduced transcription (HDAC3 and HDAC6). The HDAC family plays a critical role in development by modulating chromatin structure involved in DNA replication, repair, and gene transcription [48]. Reduced expression would lead to chromatin relaxation which can promote DNA damage and result in genomic instability [49]. Hdac3 is a key regulator of chromatin structure and inactivation triggers apoptosis in mice [50]. Additionally, Montgomery et al. found that mutant mice with a global deletion of Hdac3 resulted in early embryo lethality [51]. Hdac6 has been previously reported to be localized in the cytoplasm of germinal vesicle stage oocytes and 1-cell embryos in mice and its ectopic expression causes premature compaction of chromatin [52].

Telomeres are specialized non-coding DNA sequences located at the ends of all chromosomes that protect these regions from recombination and degradation activities as well as serve to maintain chromosome integrity [53, 54]. Telomeres shorten as a function of aging due to decreased telomerase activity leading to DNA damage that causes replicative senescence [55]. Telomerase activity is absent in most adult human tissues due to the lack of expression of TERT that is expressed in developing embryos [56, 57]. TERT expression was significantly reduced in the blastocysts from AMA women in our study, indicating reduced telomerase activity and possibly a shortening of the telomeres in this aged patient population. Telomere shortening has been shown to cause widespread changes in the transcription of genes located up to 10 Mb from a telomere in human myoblasts which could explain the high proportion of significant differentially expressed genes located in the sub-telomeric regions of AMA blastocysts [55].

Similarities were observed between our study and the publication by Kawai et al. who investigated gene expression profiles in human blastocysts relative to parental age (31–41 years of age) [23]. Several, common, downregulated genes were involved in cell growth, differentiation, and proliferation including PCBP4, PPP2R2C, MYO16, and PTPRD. Others were linked to oxidative damage, cell stress, and the inflammatory response which included MSRA, FAAP24, MICU1, and AOAH. TLE6 was also downregulated in both studies and has additionally been reported to be reduced in the trophectoderm cells from non-implanting embryos [58]. These similarities reinforce our findings that blastocysts from women of advanced maternal age are being impacted on a cell signaling level to repress the embryo’s ability to proliferate and implant.

To our knowledge, this is the first study utilizing total RNA sequencing technology to examine, specifically, the impact of advanced maternal age (≥ 42 years) on pre-implantation embryonic development directly compared to young, donor controls, highlighting the molecular changes occurring in the AMA patient. An overall compromised global transcriptome was observed in maternally aged blastocysts impacting transcriptional regulators and their biological pathways including cell growth, invasion, and an increased probability for organismal death, among others. These results provide molecular evidence of compromised embryo development with advanced maternal age, explaining the deterioration of reproductive outcomes for this patient population, independent of chromosome constitution.

## Electronic supplementary material

Supplementary Table 1 RNA sequencing alignment statistics for all samples passing quality control. (XLSX 9 kb)

Supplementary Table 2 List of significantly altered transcripts between AMA and donor control (DC) blastocysts (Q < 0.05). (XLSX 298 kb)

Supplementary Fig. 1 (A-B): Gene density for all 22 autosomes and chromosome X. Grey shaded area represents the 95th percentile, or the area where transcription changes would fall under normal chance. Differentially expressed transcripts in AMA blastocysts (orange line) that fall above or below the shaded area are considered to have a statistically higher or lower abundance in that region of the chromosome (Q < 0.05). (PNG 1018 kb)

High Resolution Image (TIF 28758 kb)

(PNG 989 kb)

High Resolution Image (TIF 28690 kb)

Supplementary Fig 2 (A-E): Top 5 predicted upstream regulators that lead to the observed differences in downstream RNA expression in AMA blastocysts vs. donor control (DC) blastocysts. Orange represents an increase and blue indicates a decrease. (A) Alpha catenin and miR-218 are predicted to be activated which inhibits downstream transcription. (B) Activation of alpha catenin along with de-activation of the NFkB complex, CCL5, and LTB4R inhibit or activate downstream transcription. (C) Activation of alpha catenin combined with de-activation of ERG, PEPL1, and the PI3K complex result in decreased downstream transcription. (D) Activation of FBN1 and de-activation of ITGA5, NRG1, CCL5, and PAX7 lead to decreased downstream transcription. (E) Activation of alpha catenin, miR-218, and SIGIRR along with de-activation of Hbb-b2 and CCL5 inhibit downstream transcription. (PNG 331 kb)

(PNG 1085 kb)

(PNG 674 kb)

(PNG 1039 kb)

(PNG 1124 kb)
