# Supplementary figures and images for: Compromised global embryonic transcriptome associated with advanced maternal age
Source: J Assist Reprod Genet. 2019 Apr 25;36(5):915–24. doi: 10.1007/s10815-019-01438-5 (PMC6541584; doi:10.1007/s10815-019-01438-5)

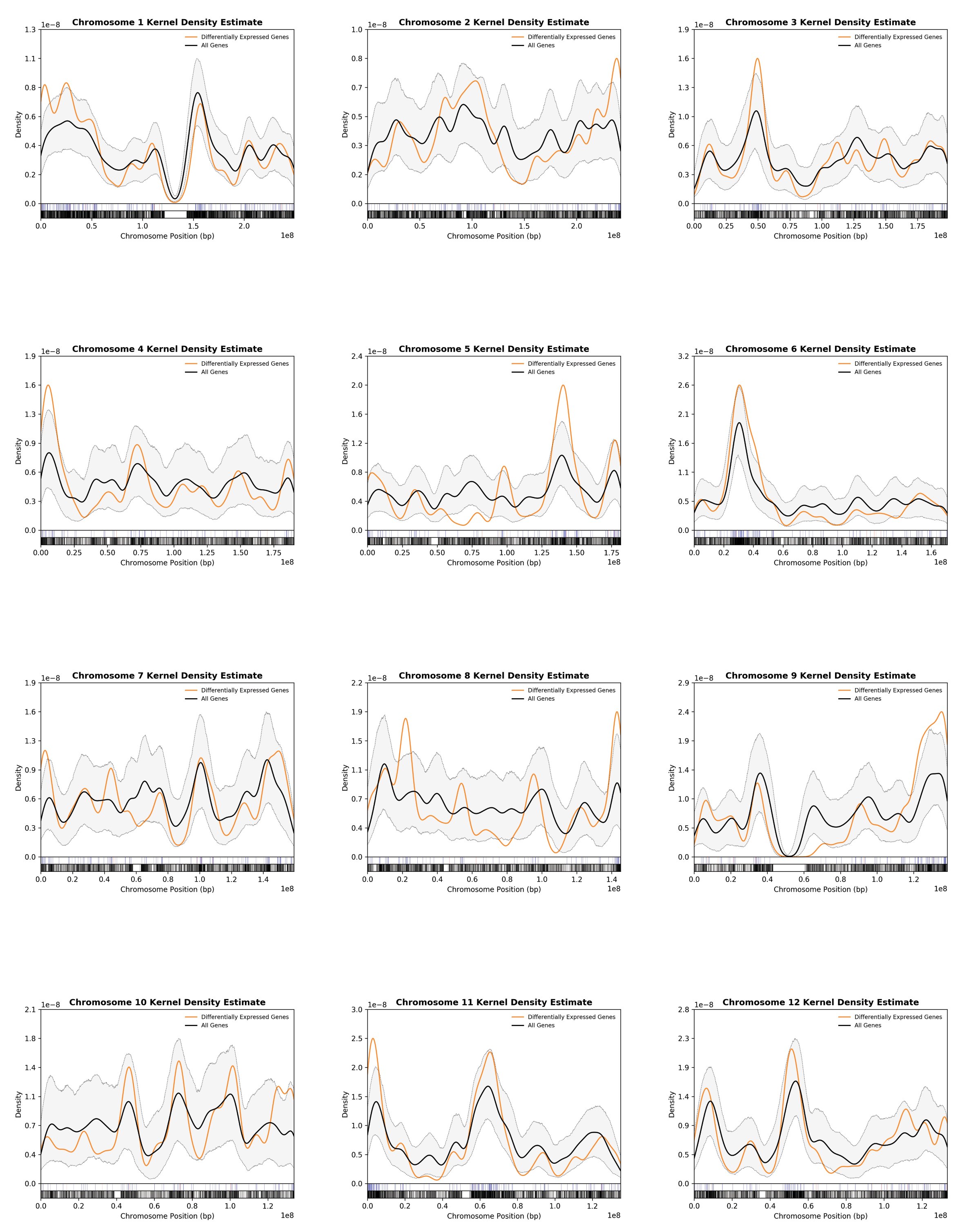

Supplement: Supplementary file 3 — (A-B): Gene density for all 22 autosomes and chromosome X. Grey shaded area represents the 95th percentile, or the area where transcription changes would fall under normal chance. Differentially expressed transcripts in AMA blastocysts (orange line) that fall above or below the shaded area are considered to have a statistically higher or lower abundance in that region of the chromosome (Q < 0.05). (PNG 1018 kb) [file 10815_2019_1438_Fig6_ESM.png]

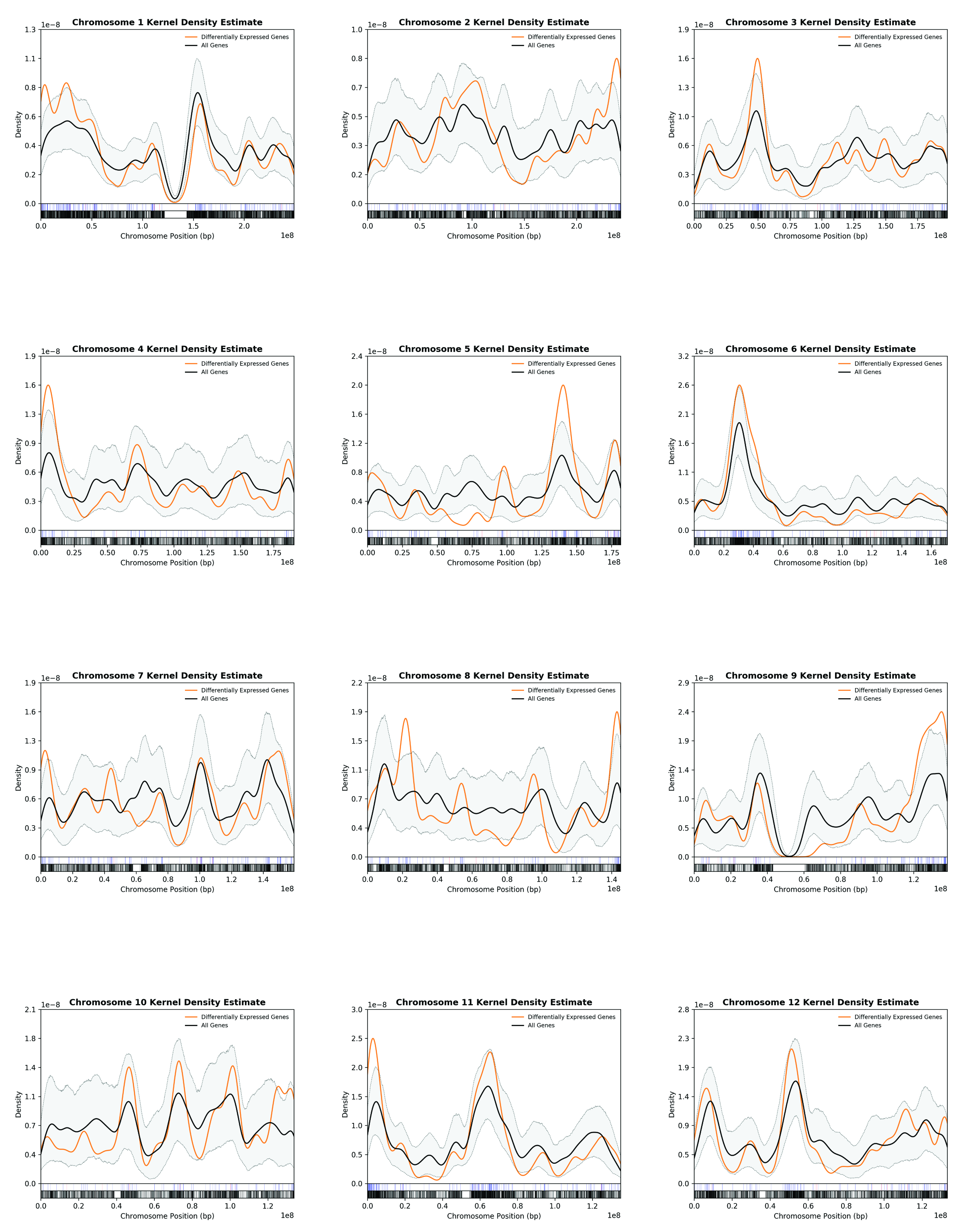

Supplement: Supplementary file 4 — High Resolution Image (TIF 28758 kb) [file 10815_2019_1438_MOESM3_ESM.tif]

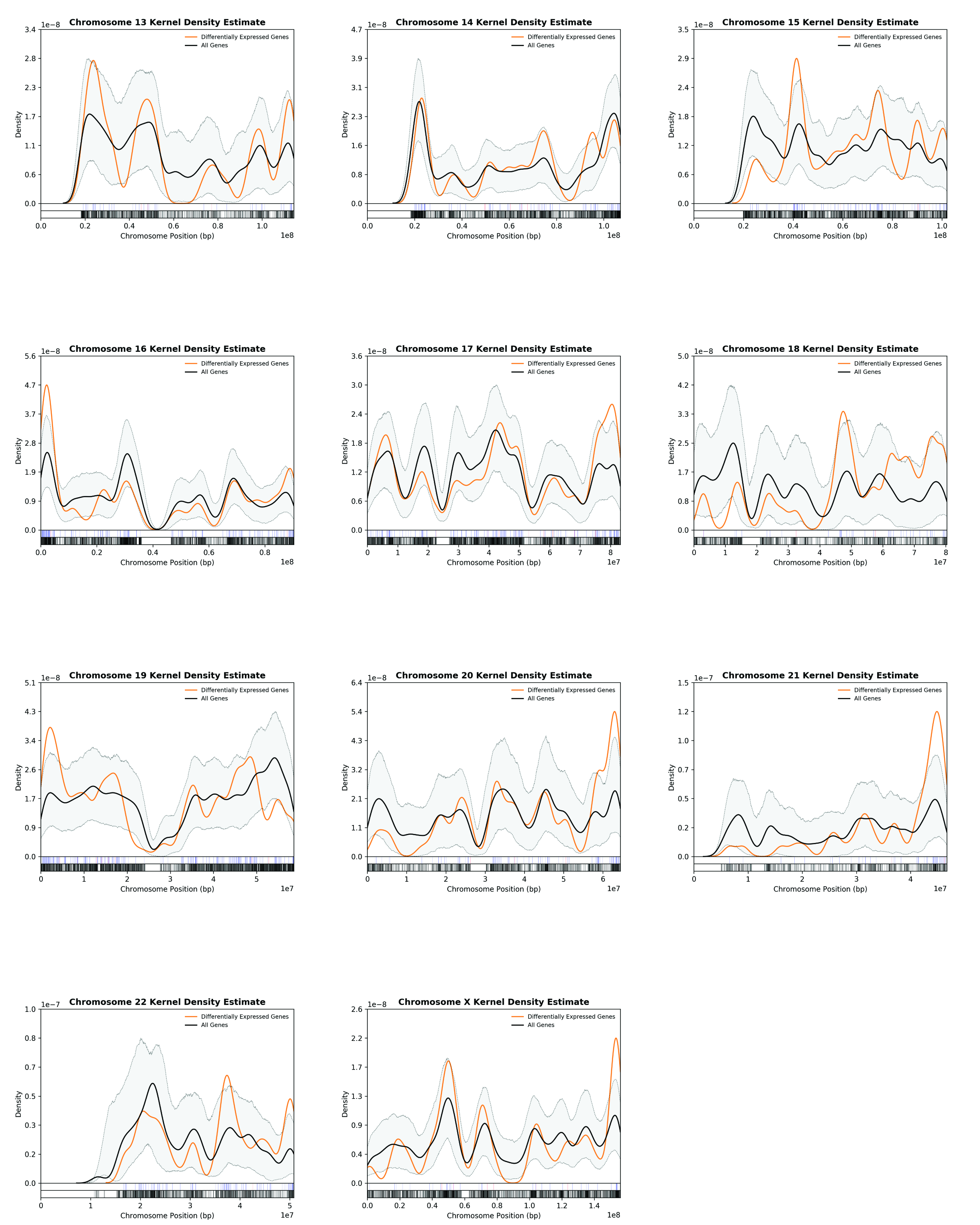

Supplement: Supplementary file 5 — (PNG 989 kb) [file 10815_2019_1438_Fig7_ESM.png]

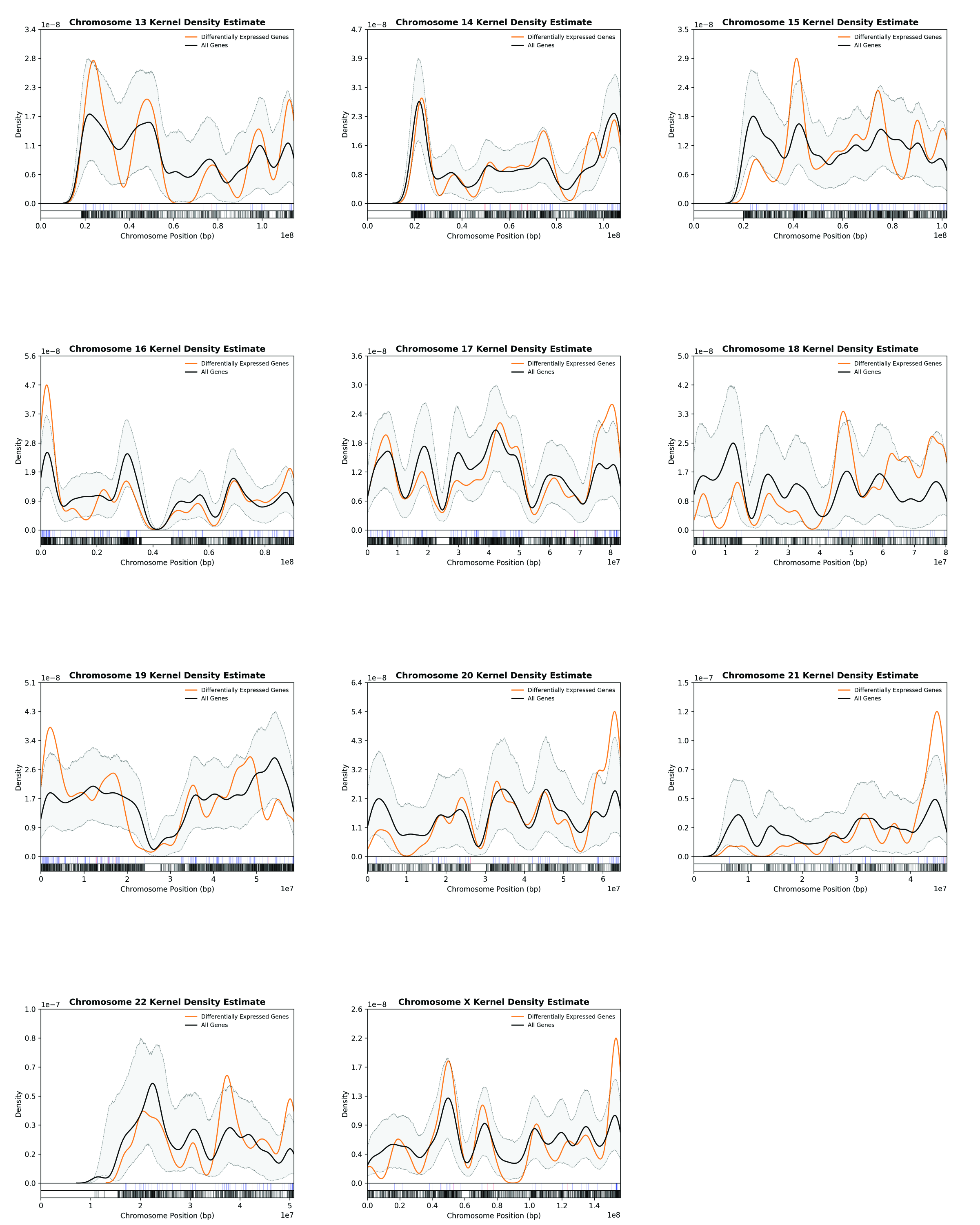

Supplement: Supplementary file 6 — High Resolution Image (TIF 28690 kb) [file 10815_2019_1438_MOESM4_ESM.tif]

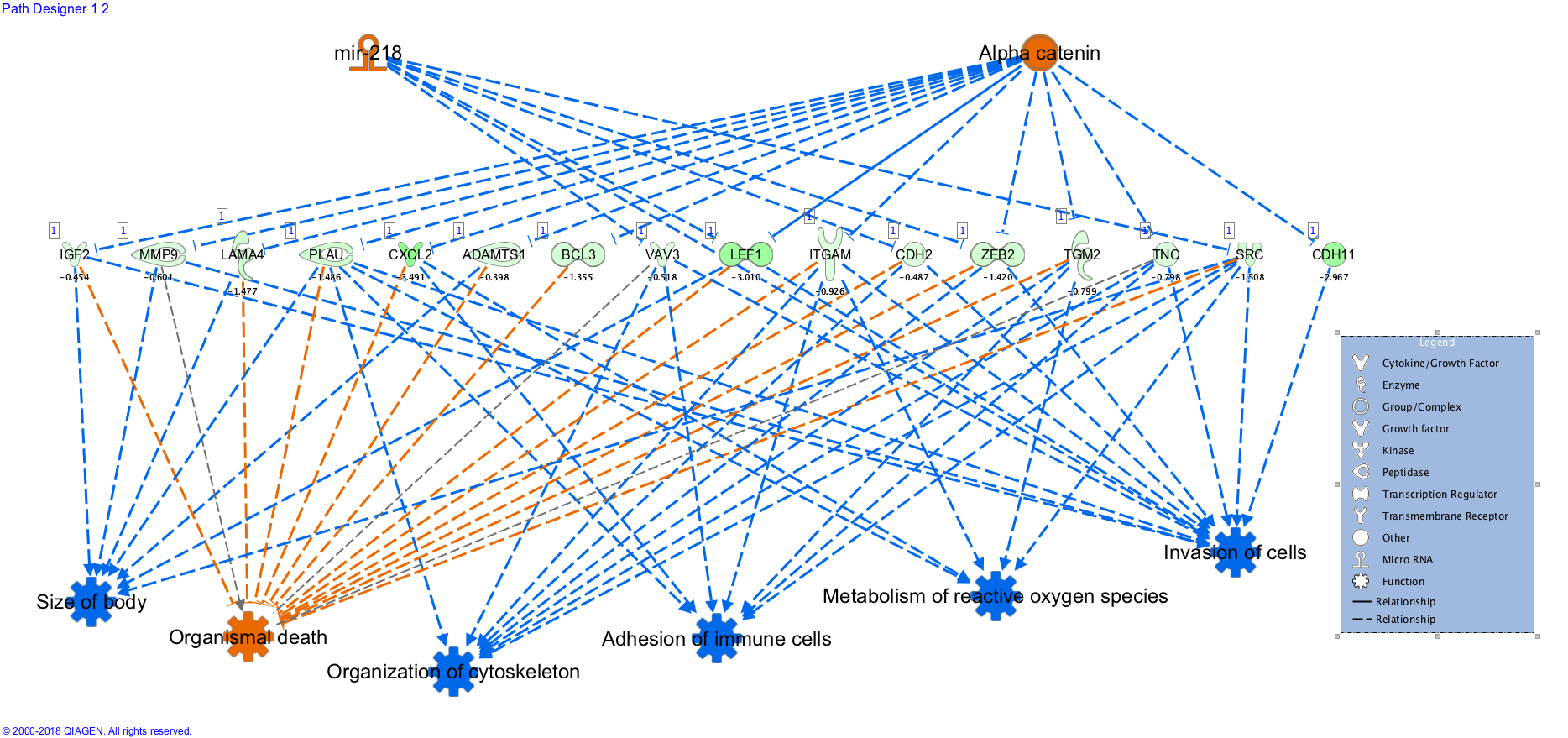

Supplement: Supplementary file 7 — (A-E): Top 5 predicted upstream regulators that lead to the observed differences in downstream RNA expression in AMA blastocysts vs. donor control (DC) blastocysts. Orange represents an increase and blue indicates a decrease. (A) Alpha catenin and miR-218 are predicted to be activated which inhibits downstream transcription. (B) Activation of alpha catenin along with de-activation of the NFkB complex, CCL5, and LTB4R inhibit or activate downstream transcription. (C) Activation of alpha catenin combined with de-activation of ERG, PEPL1, and the PI3K complex result in decreased downstream transcription. (D) Activation of FBN1 and de-activation of ITGA5, NRG1, CCL5, and PAX7 lead to decreased downstream transcription. (E) Activation of alpha catenin, miR-218, and SIGIRR along with de-activation of Hbb-b2 and CCL5 inhibit downstream transcription. (PNG 331 kb) [file 10815_2019_1438_MOESM5_ESM.png]

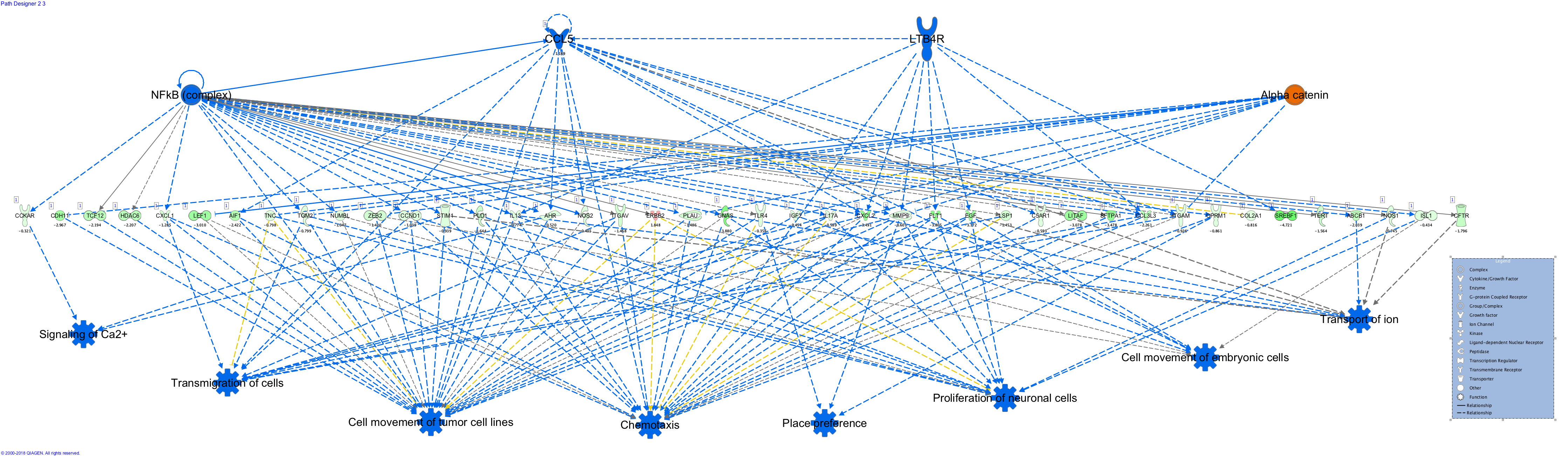

Supplement: Supplementary file 8 — (PNG 1085 kb) [file 10815_2019_1438_MOESM6_ESM.png]

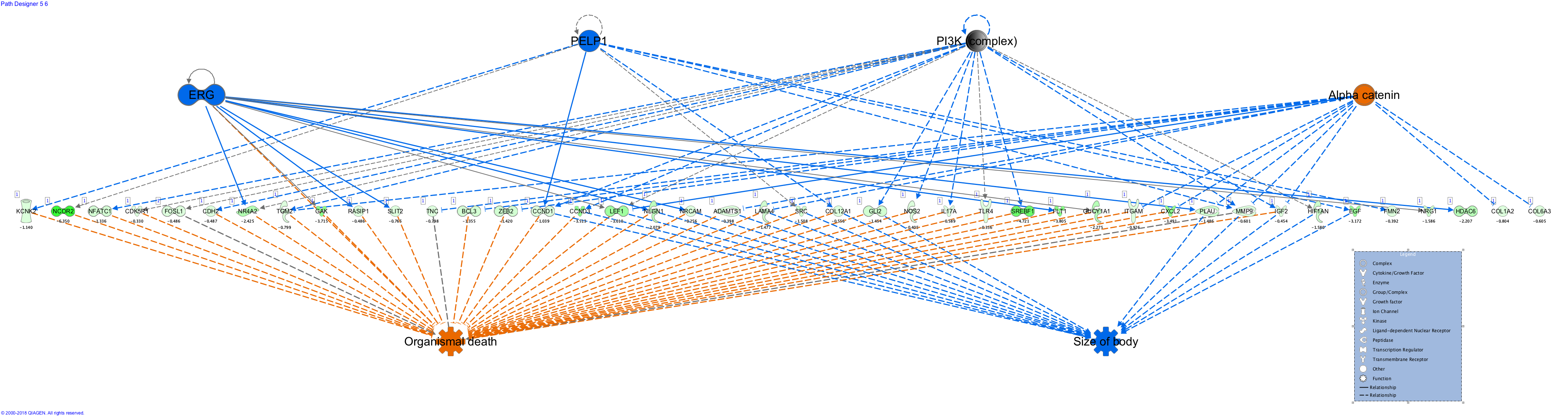

Supplement: Supplementary file 9 — (PNG 674 kb) [file 10815_2019_1438_MOESM7_ESM.png]

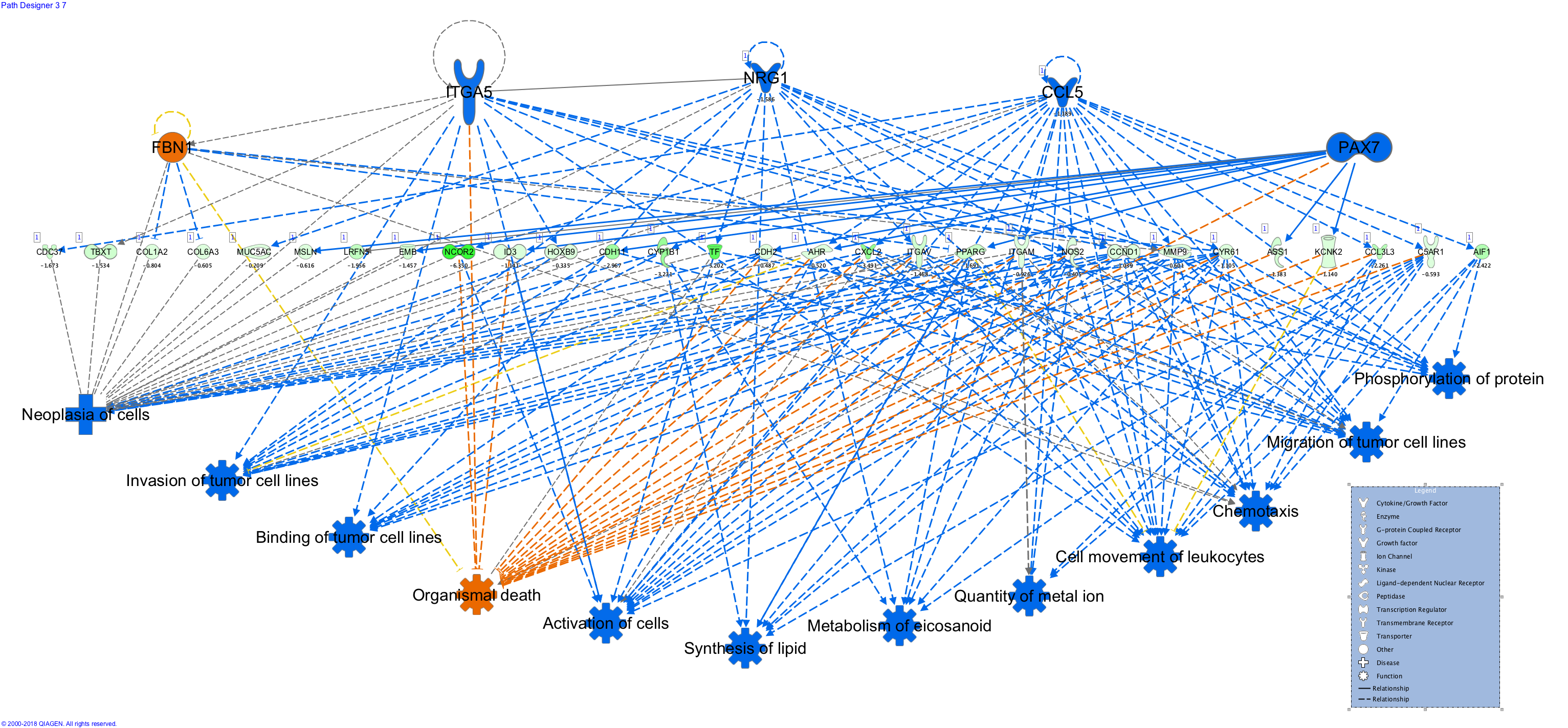

Supplement: Supplementary file 10 — (PNG 1039 kb) [file 10815_2019_1438_MOESM8_ESM.png]

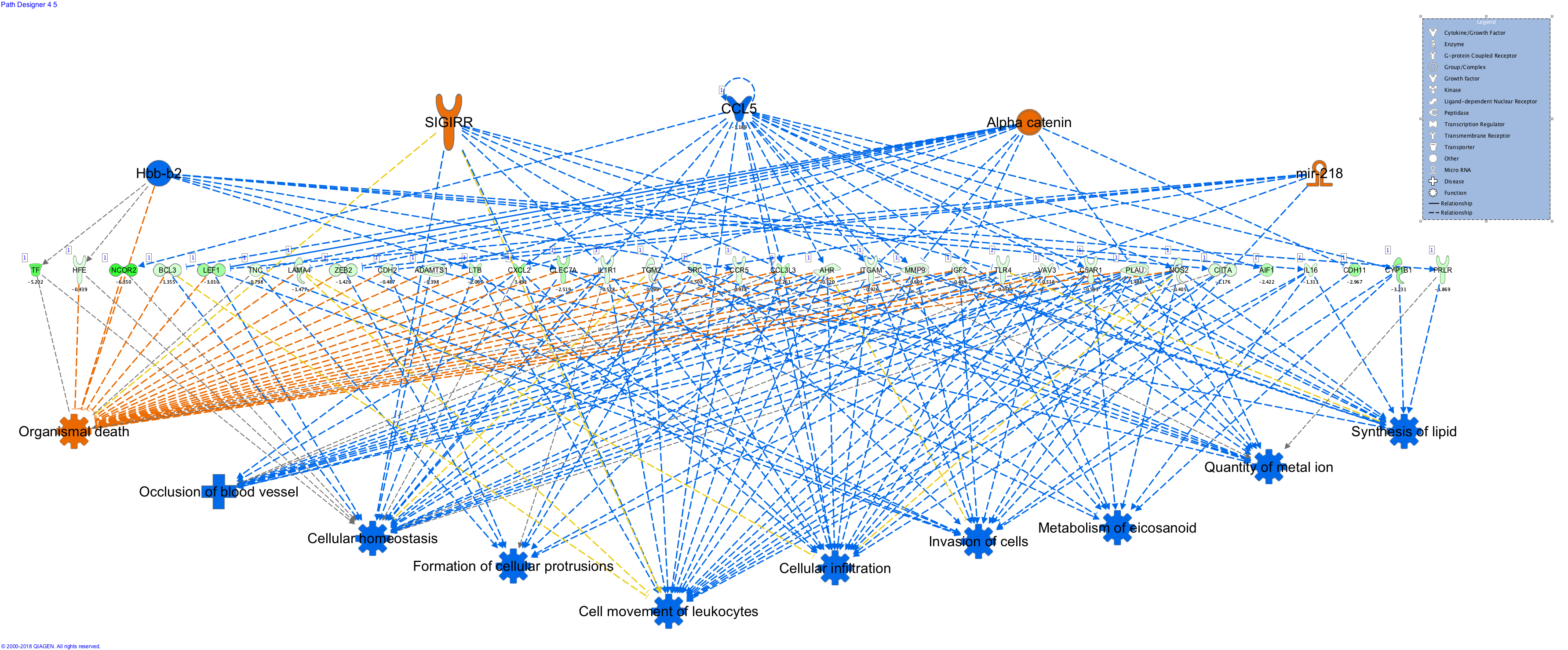

Supplement: Supplementary file 11 — (PNG 1124 kb) [file 10815_2019_1438_MOESM9_ESM.png]
